# Acute Mesenteric Ischemia as an Early Complication of COVID-19

**DOI:** 10.7759/cureus.18082

**Published:** 2021-09-18

**Authors:** Prerana Sevella, Sai Sri Harsha Rallabhandi, Vinay Jahagirdar, Shashidhar Reddy Kankanala, Akhileshwar Reddy Ginnaram, Kaanthi Rama

**Affiliations:** 1 Internal Medicine, Gandhi Hospital and Medical College, Secunderabad, IND; 2 Internal Medicine, University of Missouri Kansas City School of Medicine, Kansas City, USA; 3 Internal Medicine, Gandhi Hospital, Secunderabad, IND; 4 Internal Medicine, Gandhi Hospital and Medical College, Hyderabad, IND

**Keywords:** coronavirus, covid 19, sars-cov-2, covid coagulopathy, mesenteric ischemia

## Abstract

We herein report a case of a 44-year old male patient with coronavirus disease 2019 (COVID-19) who presented with acute mesenteric ischemia. Acute mesenteric ischemia presents with severe abdominal pain, vomiting, and constipation. The case consisted of features typical of acute mesenteric ischemia. The patient underwent laparotomy with resection of a gangrenous segment of the bowel. The radiological features of the injury along with the pathophysiology and management have been discussed.

## Introduction

Coronavirus disease 2019 (COVID-19), also called severe acute respiratory syndrome corona virus-2 (SARS-COV-2), was first identified in Wuhan, China, in December 2019. It was soon declared a pandemic on March 11, 2020 [1]. It is mainly transmitted from person to person via respiratory droplets, mucosal contact, and contaminated surfaces. Although it primarily affects the respiratory system, it can also involve the gastrointestinal, liver, cardiovascular, central nervous, and renal systems [2].

COVID-19 causes a hypercoagulable and prothrombotic state in patients [1]. It is known to cause thrombotic complications such as cerebrovascular accidents, myocardial infarction, arterial occlusion of the lower limb, splanchnic vein thrombosis, portomesenteric vein thrombosis, and mesenteric ischemia [3-7].

We present a case report describing acute mesenteric ischemia as a complication of COVID-19 infection. It is a unique case in a patient with no pre-existing comorbidities developing mesenteric ischemia.

## Case presentation

A 44-year-old male patient presented to the emergency department with a chief complaint of fever for 10 days, for which he underwent a reverse transcription-polymerase chain reaction test that was positive. He started medications prescribed by his physician, which included doxycycline 100 mg, multivitamin tablets, and paracetamol 650 mg for fever, and was placed under home quarantine according to the guidelines for his symptoms. He developed abdominal pain and constipation for three days and obstipation and vomiting for two days prior to the hospitalization. The patient has no past medical history of diabetes, hypertension, hyperlipidemia, or prior thrombotic events. He is a non-alcoholic and non-smoker.

On arrival to the emergency department, vitals were pulse rate of 130 beats per minute, regular; oxygen saturation of 90% on room air; blood pressure of 90/70 mmHg. The patient was administered crystalloids and supplemental oxygen at 4 liters/min. Screening for COVID-19 was done using a rapid antigen kit and was found to be positive. It was followed by a reverse transcription-polymerase chain reaction (RT-PCR), which was positive as well.

On physical examination, the abdomen was distended with diffuse tenderness. No guarding or rigidity was noted. Bowel sounds were absent. Digital rectal examination revealed a normal sphincter tone with the collapsed rectum and absent fecal stain on the gloved finger. Nasogastric tube decompression and Foley catheterization were done.

Table 1 lists routine blood investigations at the time of admission.

**Table 1:** Laboratory values INR: international normalized ratio; PCo2: partial pressure of carbon dioxide; PaO2: partial pressure of oxygen; HCO3: bicarbonate

Parameter	Value	Normal
Hemoglobin	14g/dl	13.5 to 17.5g/dl
Leukocyte count	23,400/ul	4,500 to 11,500/ul
Platelet count	360,000/mm3	150,000 to 450,000/mm3
Creatinine	1.4 mg/dl	0.6 to 1.2 mg/dl
Blood urea	77 mg/dl	7-18 mg/dl
Sodium	133 mEq/L	136-146 mEq/L
Potassium	5.1 mEq/L	3.5 to 5.0 mEq/L
Chloride	99 mEq/L	95 to 105 mEq/L
Lactate dehydrogenase	1097 U/L	45 to 200 U/L
D-dimer	1590 ng/mL	<250 ng/mL
Prothrombin time	17 seconds	11-15 seconds
INR	1.44	<1.1
Parameter	Value	Normal
pH	7.409	7.35-7.45
PCo2	21.8 mm Hg	33-45 mm Hg
PaO2	99.8 mm Hg	75-105 mm Hg
HCO3	13.5 mEq/L	22-28 mEq/L

Certain tests like gamma-glutamyl transferase and amylase could not be performed due to the non-availability of the tests in the hospital on an emergency basis. After stabilizing the patient hemodynamically, he underwent an ultrasound of the abdomen and a computed tomography scan of the chest and abdomen. Ultrasound of the abdomen showed prominent small bowel with an increased caliber and absent peristalsis with to and fro movements of contents. Moderate ascites was noted.

**Figure 1:**
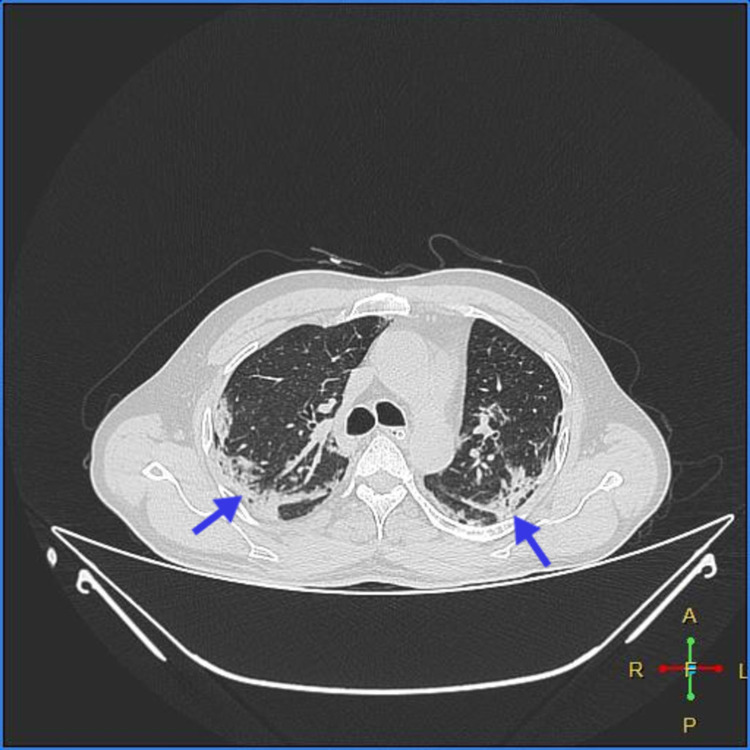
Multiple, patchy, irregular, ground-glass opacities with interstitial thickening in bilateral lung fields with peripheral, subpleural, and posterior predominance

**Figure 2:**
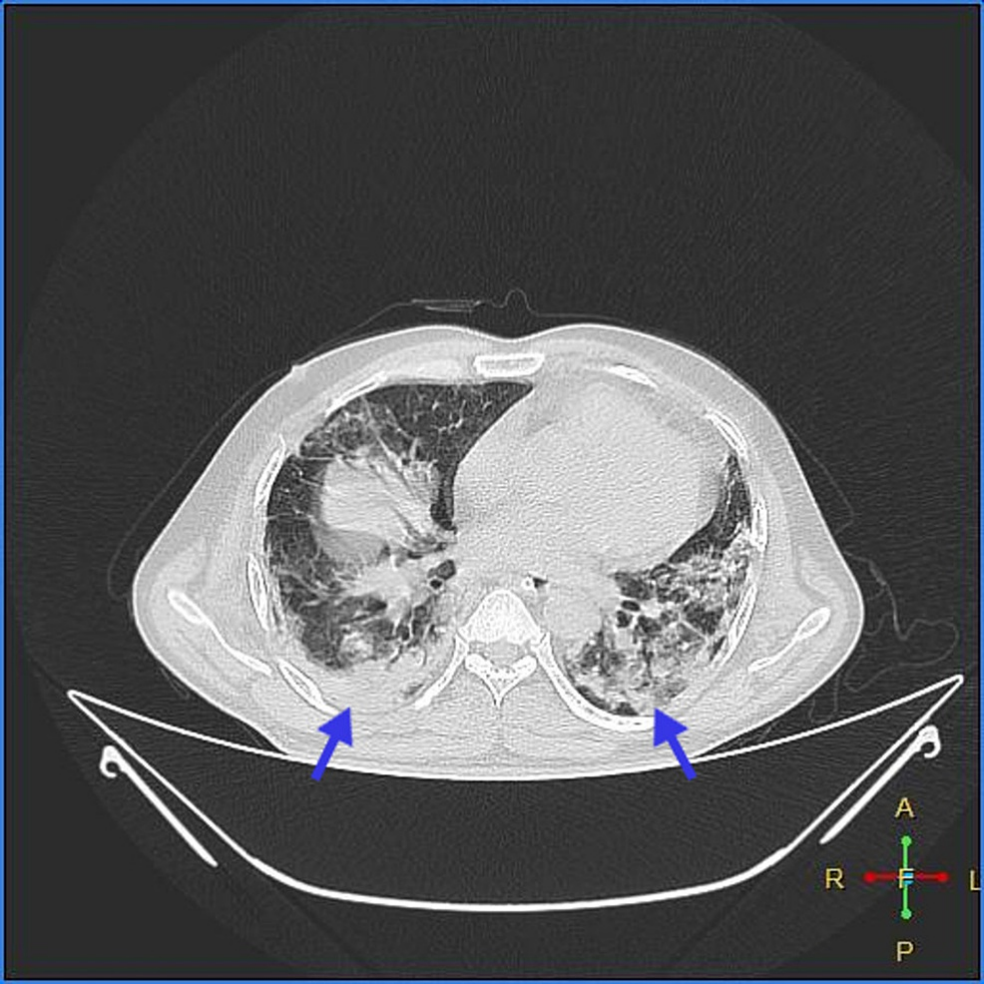
Multiple, patchy, irregular, ground-glass opacities with interstitial thickening in bilateral lung fields with peripheral, subpleural, and posterior predominance

As shown in Figures 1-2, high-resolution CT of the chest showed multiple patchy irregular ground-glass opacities with interstitial thickening in bilateral lung fields with peripheral, subpleural, and posterior predominance, suggestive of atypical viral pneumonitis The Coronavirus Disease 2019 Reporting and Data System (CORADS)-5, computed tomography severity index (CTSI)-14/25-MODERATE).

**Figure 3:**
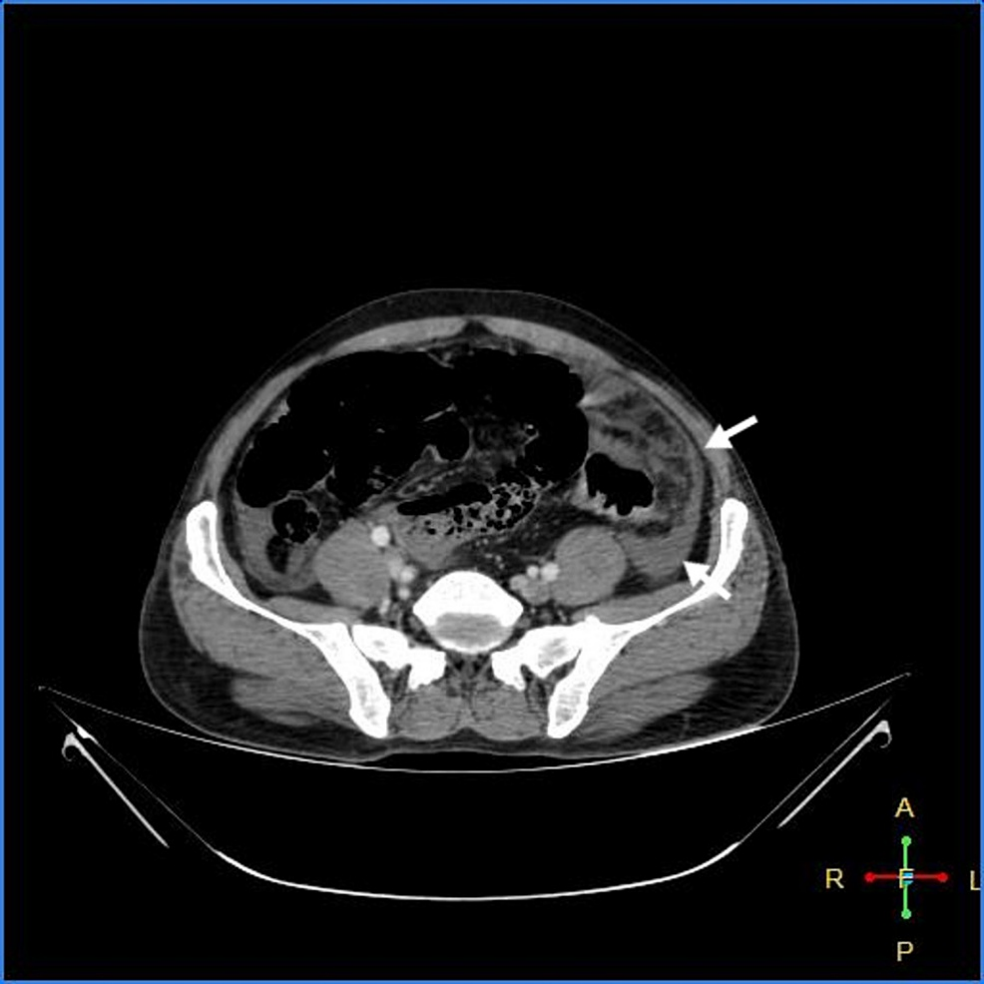
Peritoneal thickening with fat stranding

**Figure 4:**
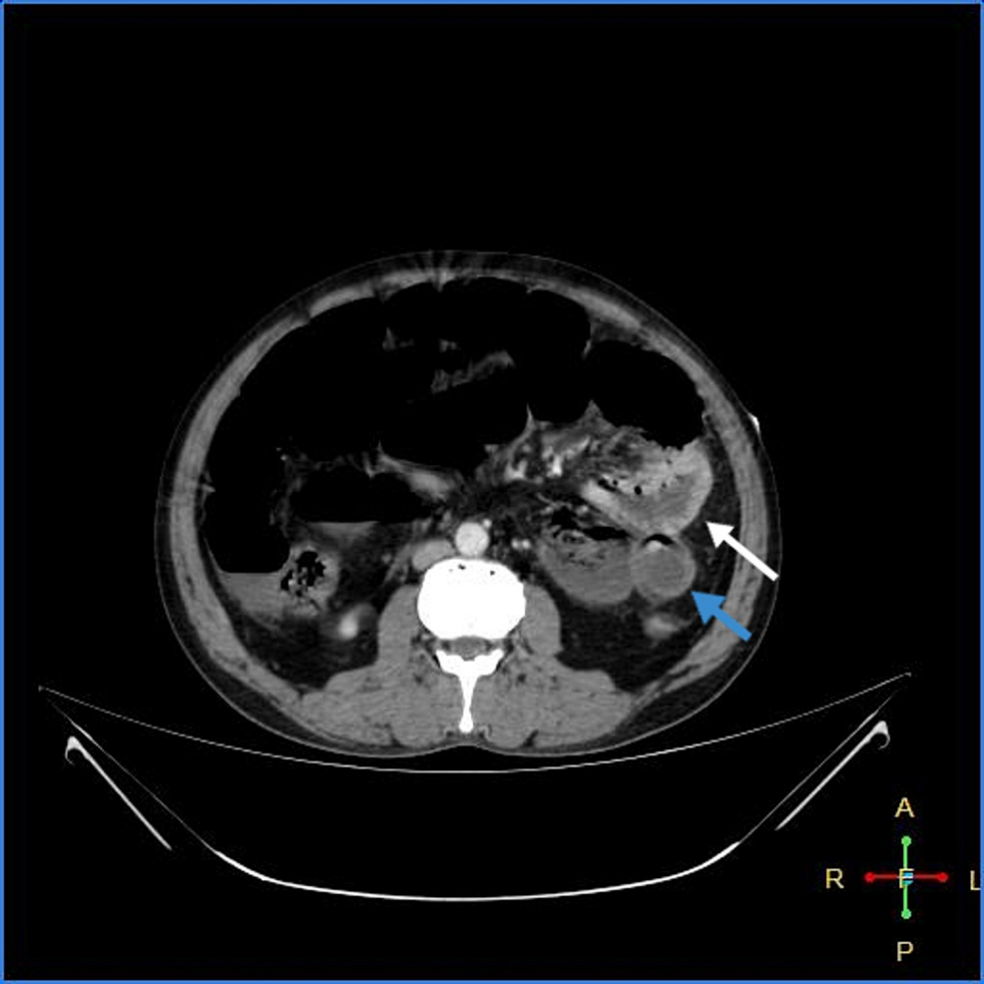
Viable jejunum (white arrow); ischemic bowel (blue arrow)

**Figure 5:**
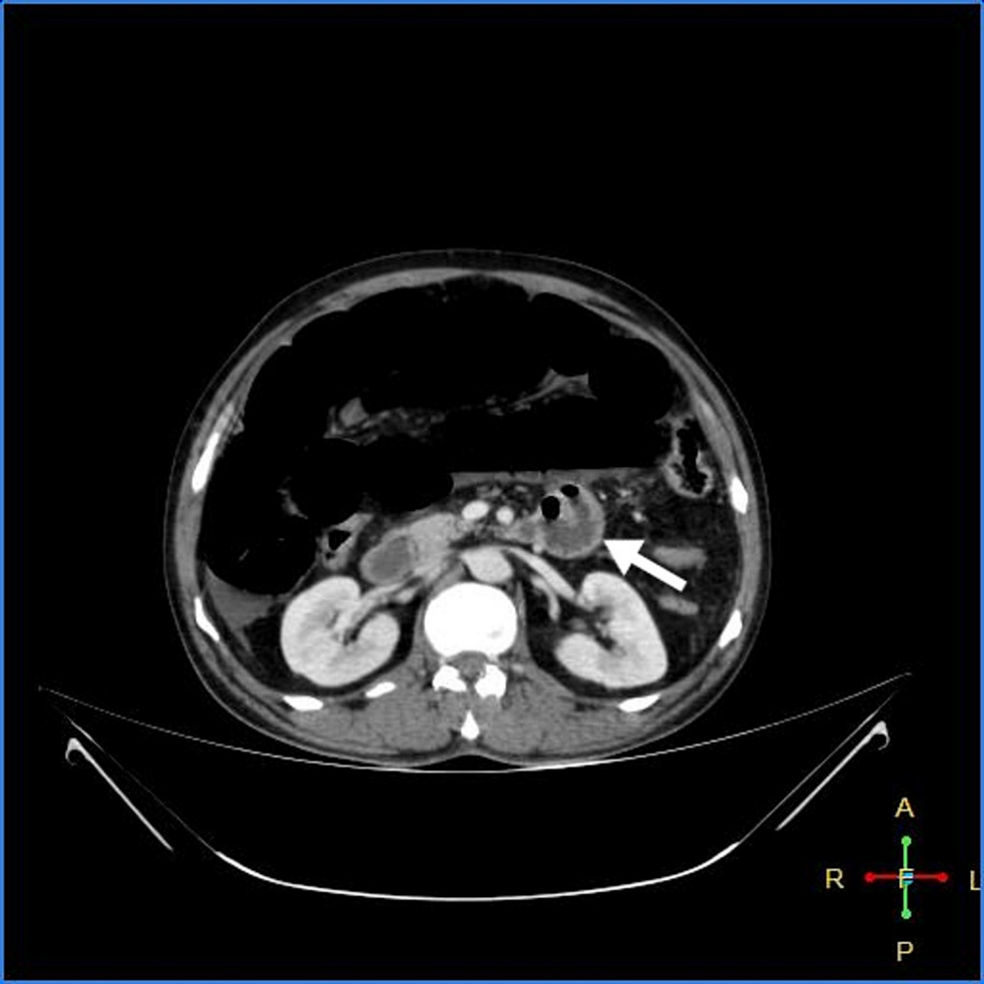
Viable jejunum

**Figure 6:**
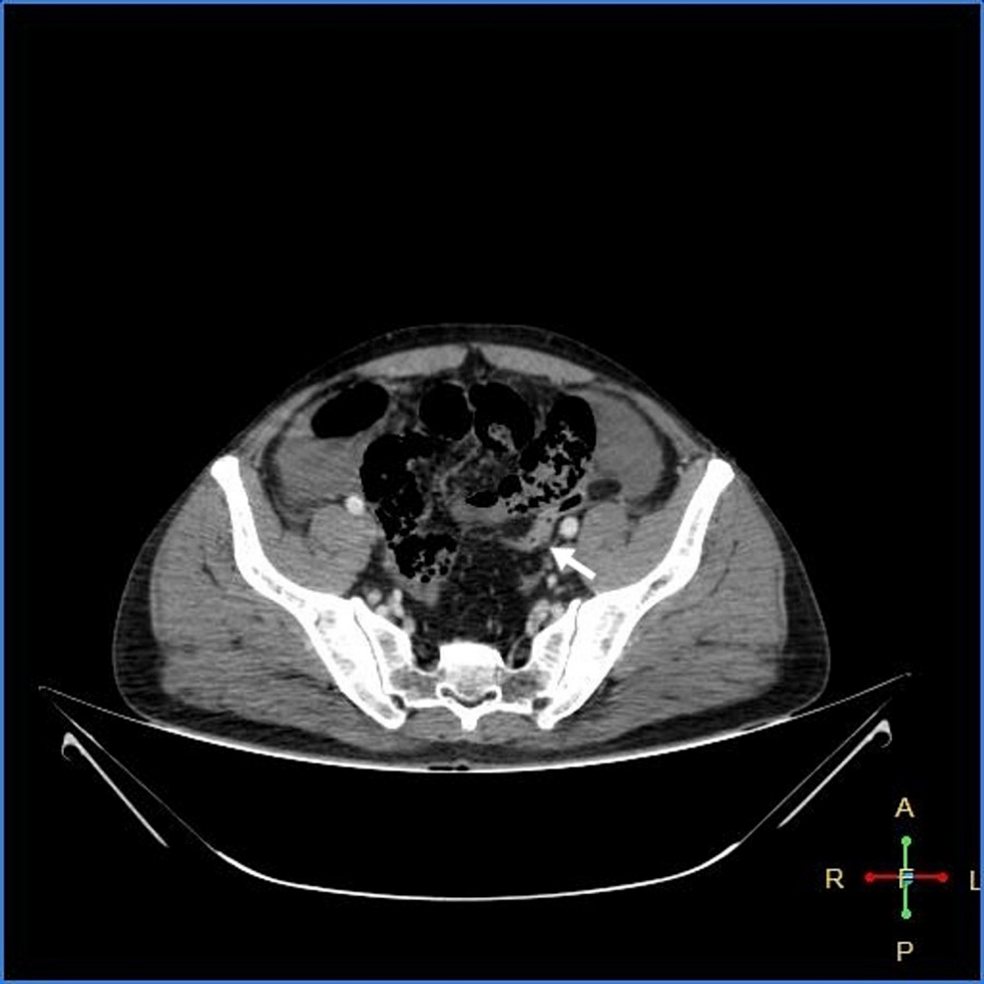
Collapsed sigmoid colon

**Figure 7:**
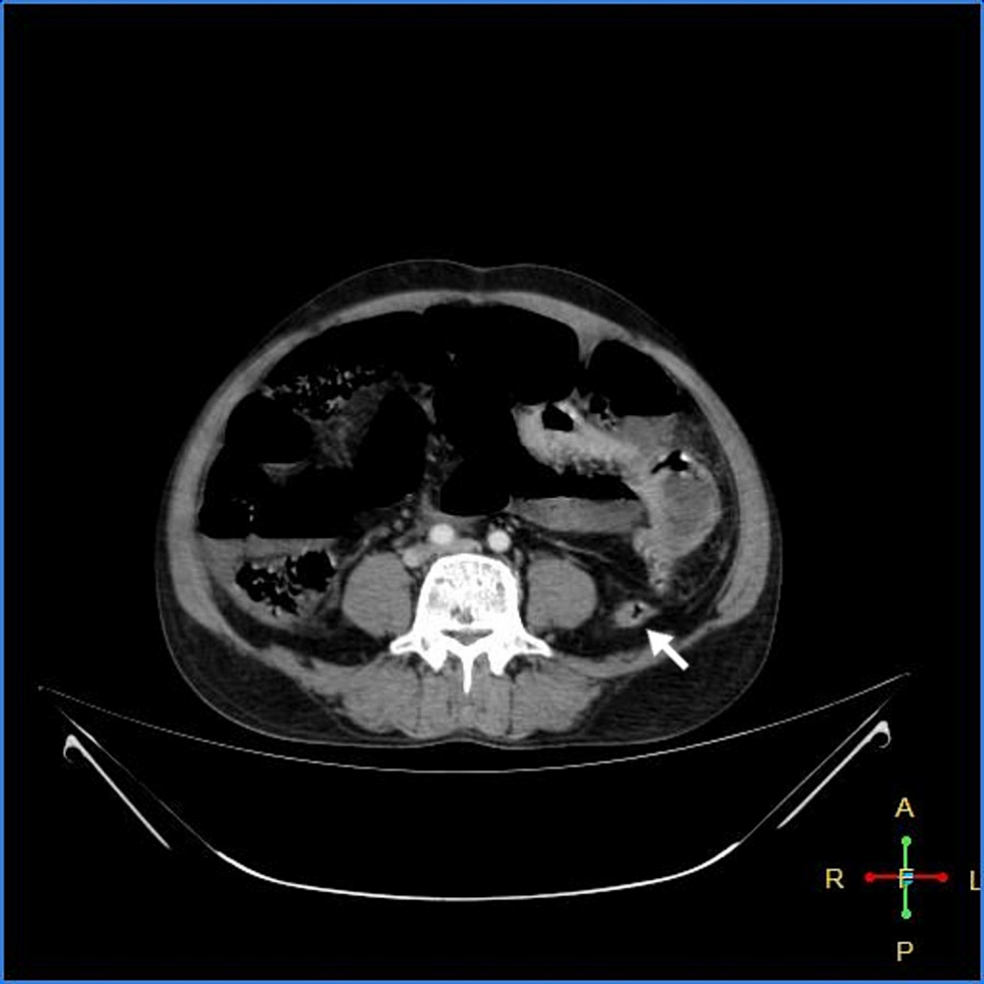
Collapsed descending colon

As shown in Figures 3-7 below, CT abdomen showed findings suggestive of segment contrast-enhanced (viable) small bowel with adjacent fat stranding. The collapse of the transverse colon, hepatic flexure, descending colon, and sigmoid colon was noted. Moderate ascites was present, with thickening of the peritoneum with mesenteric fat stranding in the left lumbar region and left iliac fossa - signs of peritonitis.

**Figure 8:**
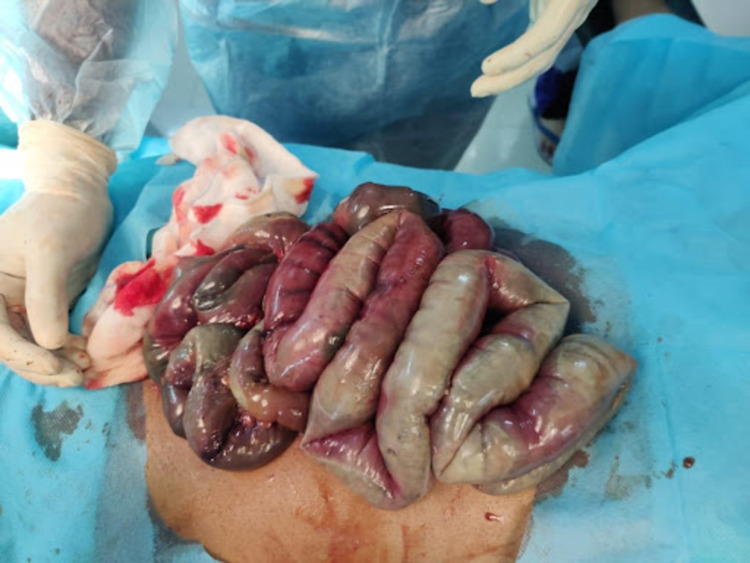
Gangrenous bowel

Considering mesenteric ischemia as the probable diagnosis, the patient was shifted to the emergency operating room and proceeded with laparotomy after administering one dose of piperacillin 4 g with tazobactam IV 500 mg. The intraoperative findings shown in Figure 8 were gangrenous bowel from 10 cm distal to the duodenojejunal flexure up to the mid-transverse colon.

Resection of the gangrenous segment of the bowel with end jejunostomy and mucus fistula of the transverse colon was done. The patient was then shifted to the surgical intensive care unit without extubating. He received piperacillin 4 g with tazobactam IV 500 mg thrice daily and metronidazole IV 500 mg thrice daily along with low molecular weight heparin 60 mg twice daily. During the second postoperative day, the patient desaturated and passed away due to a cardiopulmonary arrest.

## Discussion

The COVID-19 pandemic has spread to more than 200 countries. Along with the predominant involvement of the respiratory system, involvement of other systems is becoming more evident. Thrombosis of the venous and arterial vessels is a known complication, especially in severely ill patients. Male gender, hypertension, obesity, cardiovascular disease, and prolonged immobilization are the main risk factors for thrombotic complications [8]. There is an increasing number of cases being reported in patients with no contributing risk factors or comorbidities [9]. Coagulopathy affects the micro-vascular beds like the pulmonary, renal, and hepatic systems, leading to end-organ damage, and the macrovascular large vessels, leading to deep vein thrombosis (DVT), stroke, myocardial infarction, lower limb ischemia, and mesenteric ischemia [10].

Acute mesenteric ischemia (AMI) is a life-threatening complication caused by inadequate blood flow through the mesenteric vessels, resulting in ischemia and eventual gangrene of the bowel wall. The exact mechanism by which SARS-COV-2 causes AMI is unknown, but several hypotheses have been described. SARS-COV-2 causes AMI by both direct and indirect mechanisms. It contains a spike protein called protein S on its membrane that can bind to the angiotensin-converting enzyme (ACE2) receptor located on the membranes of host cells. The lungs, intestine, oral, mucosa, liver, and endothelium are rich in ACE2 receptors [8]. The binding of the virus to the ACE2 receptors causes endothelial injury, leading to activation of the tissue factor pathway, which causes increased thrombin generation and fibrin formation. The typical finding in patients with COVID-19 is an increase in procoagulants, seen as an increase in prothrombin time, fibrinogen, and D-dimer, indicating a hypercoagulable state with a decrease in platelet count and near-normal activated partial thromboplastin (APTT) [3]. In our case, we observed an increase in prothrombin time (PT), D-dimer, and international normalized ratio (INR) with normal platelet count. Another possible contributing factor for thromboembolism in COVID-19 patients may be the hypoxia associated with severe COVID-19. Hypoxia stimulates thrombosis by increasing the blood viscosity and hypoxia-inducible transcription factor signaling pathway [9,11]. Along with the endothelial injury and hypercoagulability described above, stasis is often seen in critically ill COVID-19 patients because of prolonged bed rest and immobilization in the critical care unit. Therefore, COVID-19 predisposes patients to a thromboembolic state by fulfilling Virchow's triad, which includes endothelial injury, stasis of blood flow, and hypercoagulability [1].

AMI should be suspected in patients presenting with gastrointestinal symptoms like nausea, vomiting, abdominal pain out of proportion to the examination, diarrhea, and abdominal distension. Laboratory investigations must include prothrombin time, ferritin, lactate, and D-dimer, as they are often deranged and may aid in diagnosis. CT angiography is the diagnostic test of choice [1]. However, we did not perform a CT angiography due to the rapid deterioration of the patient, and the CT scan provided enough evidence of bowel necrosis.

Treatment includes early fluid resuscitation, use of broad-spectrum antibiotics, and surgical resection of the necrotic bowel, along with restoration of blood supply. Hemodynamic resuscitation and gastrointestinal decompression are often required [11-12]. Emergency laparotomy must be performed immediately after the onset of abdominal pain to achieve a favorable outcome [11]. Patients with COVID-19 have a high rate of mortality post-surgery, even if the surgery is performed during the incubation period, which is one to 14 days following contact with an infectious source. The mortality rate in patients undergoing emergency surgeries is approximately 25.6% [1,13].

Thromboprophylaxis is recommended for all hospitalized COVID-19 patients. Low molecular weight heparin must be used at the appropriate dose depending on the comorbidities and severity of the disease [1]. The doses recommended are based on patient factors, including creatinine clearance, body mass index (BMI), and the presence of pre-existing risk factors.

**Table 2:** Creatinine clearance and LMWH dose recommended LMWH: low molecular weight heparin

Creatinine clearance	LMWH dosage recommended
>30 mL/min	40 mg daily
15-30 mL/min	30 mg daily

Patients with a creatinine clearance of <15 mL/min must receive unfractionated heparin. If BMI is >40 kg/m^2^, the dose must be increased by 30%. It is recommended to continue anti-coagulants for at least three months after hospital discharge in patients with risk factors for thrombotic complications. They must also be monitored periodically for changes in prothrombin time, platelet counts, and D-dimer levels. Alternatives to heparin such as factor Xa inhibitor drugs (rivaroxaban, betrixaban) may be used long-term [1].

## Conclusions

A high index of suspicion is required in patients with COVID-19, to prevent and manage thrombotic complications effectively. Coagulation profiles must be monitored regularly. Thrombotic complications can occur in any COVID-19 positive patients, even in the absence of risk factors or comorbidities.
